# Using the sociotechnical model to conduct a focused usability assessment of a breast reconstruction decision tool

**DOI:** 10.1186/s12911-023-02236-x

**Published:** 2023-07-28

**Authors:** Randi Foraker, Crystal Phommasathit, Kaleigh Clevenger, Clara Lee, Jessica Boateng, Napiera Shareef, Mary C. Politi

**Affiliations:** 1 Division of General Medical Sciences, Department of Internal Medicine, Washington University in St. Louis School of Medicine, Saint Louis, MO USA; 2 Comprehensive Cancer Center, College of Health Sciences, The Ohio State University, Columbus, OH USA; 3 Department of Plastic and Reconstructive Surgery, College of Medicine, Division of Health Services Management and Policy, College of Public Health, The Ohio State University, Columbus, OH USA; 4 Division of Public Health Sciences, Department of Surgery, School of Medicine, Washington University in St Louis, Saint Louis, MO USA; 5 College of Medicine, The Ohio State University, Columbus, OH USA

**Keywords:** Breast cancer, Breast reconstruction, Clinical decision aid, Sociotechnical framework

## Abstract

**Introduction:**

BREASTChoice is a web-based breast reconstruction decision aid. The previous clinical trial—prior to the adaptation of this refined tool in which we explored usability—measured decision quality, quality of life, patient activation, shared decision making, and treatment choice. The current usability study was designed to elicit patients’ and clinicians’ perspectives on barriers and facilitators for implementing BREASTChoice into the clinical workflow.

**Methods:**

We conducted qualitative interviews with patients and clinicians from two Midwestern medical specialty centers from August 2020 to April 2021. Interviews were first double coded until coders achieved a kappa > 0.8 and percent agreement > 95%, then were coded independently. We used a sociotechnical framework to evaluate BREASTChoice’s implementation and sustainability potential according to end-users, human-computer interaction, and contextual factors.

**Results:**

Twelve clinicians and ten patients completed interviews. Using the sociotechnical framework we determined the following. People Using the Tool: Patients and clinicians agreed that BREASTChoice could help patients make more informed decisions about their reconstruction and prepare better for their first plastic surgery appointment. Workflow and Communications: They felt that BREASTChoice could improve communication and process if the patient could view the tool at home and/or in the waiting room. Clinicians suggested the information from BREASTChoice about patients’ risks and preferences be included in the patient’s chart or the clinician electronic health record (EHR) inbox for accessibility during the consultation. Human Computer Interface: Patients and clinicians stated that the tool contains helpful information, does not require much time for the patient to use, and efficiently fills gaps in knowledge. Although patients found the risk profile information helpful, they reported needing time to read and digest.

**Conclusion:**

BREASTChoice was perceived as highly usable by patients and clinicians and has the potential for sustainability. Future research will implement and test the tool after integrating the stakeholder-suggested changes to its delivery process and content. It is critical to conduct usability assessments such as these prior to decision aid implementation to ensure success of the tool to improve risk communication.

**Supplementary Information:**

The online version contains supplementary material available at 10.1186/s12911-023-02236-x.

## Introduction

Clinical decision support (CDS) tools can facilitate point-of-care decision-making, particularly when they are thoughtfully designed to be user-centered and maximize principles of human-computer interaction [1, 2]. These types of tools, planned as both patient- and clinician-facing, have been successfully tested to improve a broad range of health outcomes [2, 3]. Breast reconstruction surgery restores the breast shape after mastectomy and can be performed at the time of mastectomy (immediate reconstruction) or months to years later (delayed reconstruction). Breast reconstruction can restore quality of life after mastectomy, but the risk of complications is relatively high. Many patients do not understand the risks and tradeoffs of the procedure, and decisions are often misaligned with patient preferences.

The previous clinical trial—prior to the adaptation of this refined tool in which we explored usability—measured decision quality, quality of life, patient activation, shared decision making, and treatment choice. In the prior study, the tool was tested as a website that patients logged into on their home computers or in clinic. Our previous work has demonstrated the efficacy of a CDS tool to support women’s decisions about post-mastectomy breast reconstruction (BREASTChoice) [4]. BREASTChoice is a web-based breast reconstruction decision aid that incorporates personalized risk estimates using data from the electronic health record (EHR), education about the pros and cons of breast reconstruction options, and a clinician summary to review at the point-of-care [5].

In response to feedback from the earlier trial and a stakeholder advisory board, preliminary work and tool adaptation included integrating photos, and improving the layout, flow of the risk page, and order of the information to ensure that they were patient-centered and relatable to users. A follow-up study evaluated factors that could impact implementation of the BREASTChoice tool according to patients, clinicians, and informatics professionals [4]. Stakeholders reported that BREASTChoice had the potential to facilitate shared decision-making, improve workflow, and enhance the efficiency of a breast reconstruction consultation. Prior to implementation of BREASTChoice in routine clinical care, stakeholders suggested exploring the function and use of particular features and factors which make the CDS tool conducive to use and sustainable. This study set out to test the usability of BREASTChoice in two settings with diverse patient populations.

Our overall objective is to implement the BREASTChoice tool in two academic medical centers for use among patients and clinicians. The current usability study was designed to elicit barriers and facilitators to ease the process of implementation and incorporate the tool into the clinical workflow from the perspective of patients and clinicians.

## Methods

We conducted qualitative interviews with patients and clinicians from two Midwestern medical specialty centers and used a sociotechnical framework to evaluate BREASTChoice implementation and sustainability potential according to end-users, human-computer interaction, and contextual factors. The sociotechnical framework we employed has been used in our previous studies [6]. We hypothesized that we would identify modifiable factors to the workflow and content that would improve the tool’s utility and sustainability. All methods were carried out in accordance with relevant guidelines and regulations. All experimental protocols were approved by institutional review boards. Participants provided informed consent.

### Conceptual framework

**Fig. 1:**
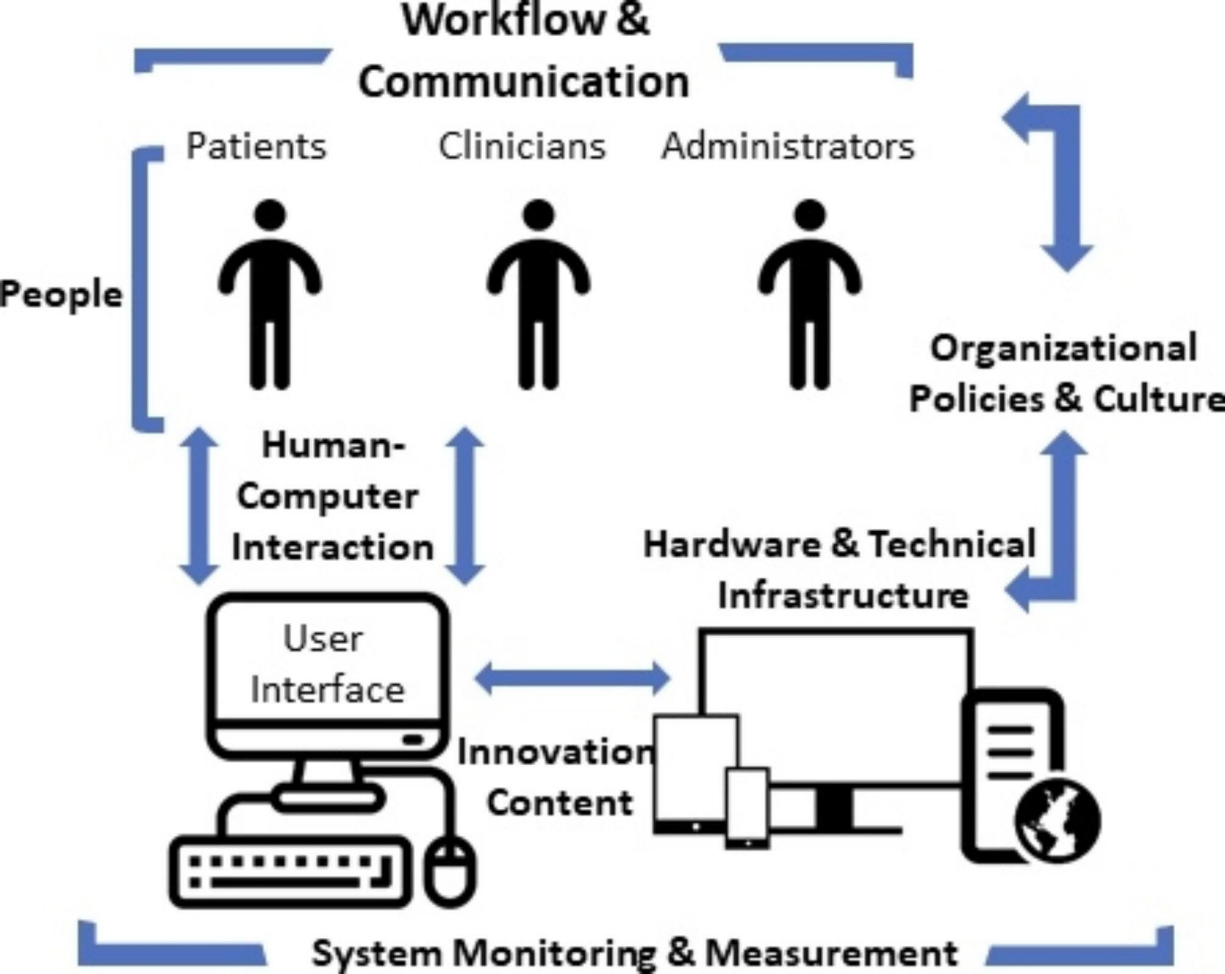
Adapted sociotechnical framework

Using inductive thematic analysis, we adapted the sociotechnical framework (Fig. 1) as a guide to develop the codebook [6]. Framework constructs comprised the following: (1) people (participants, patients and clinicians); (2) workflow and communication (participant opinions regarding the timing of tool delivery and summary of tool content); (3) organizational policies and culture (participant perspectives on EHR integration of tool); (4) hardware and technical infrastructure (participant perceptions of utility of tablet, home, or clinic computers for delivery of the tool); (5) innovation content (participant views about tool content); (6) human-computer interaction (participant feedback regarding tool duration, and ease of navigating the tool); and (7) system monitoring and measurement (factors associated with sustained use of the tool).

### Study population

Eligible clinicians included reconstructive surgeons, reconstructive surgery physician assistants, surgical oncologists, and patients of the reconstructive surgery department. Eligibility criteria for patients were English-speaking women over the age of 18 with a history of Stage I-III ductal or lobular carcinoma or ductal carcinoma in situ (DCIS), treated with mastectomy within the last five years were eligible to participate. Women who did not have mastectomy or had a diagnosis of a histology type besides ductal or lobular carcinoma or DCIS were excluded from participation. Women who had stage IV disease at the time of surgery or were being treated by institutions outside of the implementation sites were also excluded.

The rationale for recruiting women who had been through this decision-making process within recent years was to obtain their detailed perspective on the process while not adding burden to their healthcare and decision-making experience. We chose to group patients together regardless of type of reconstruction, since our study’s primary objective was to evaluate usability of the tool as opposed to the content of the tool. If a woman underwent two-stage implant reconstruction or tissue-expander surgery and then flap reconstruction, they were included in the immediate cohort. We chose not to include the names of the study sites for confidentiality purposes due to the relatively small number of clinicians employed at each site and thus eligible for inclusion.

### Data collection

We created a semi-structured interview guide for patients and clinicians (see Appendix). We did not change the interview guide during the course of the study, but had asked our advisory board to review it prior to its use in the current study. We designed the interview questions to engage participants in a think-aloud format to get real-time feedback as they navigated through the website.

On the day of the interview, participants were sent a link to BREASTChoice, which was housed on a website outside the EHR. We explained the purpose of the study, and participants completed an informed consent or a waiver of informed consent. Interviews were conducted by masters-level research coordinators virtually and video recorded using Zoom between August 2020 and April 2021. Interviewers were trained and supervised by the principal investigators of the study (M.P., C.L.), both of whom have experience with qualitative interviewing and analysis. Interviews each lasted about 20–40 min, and field notes were taken during each session. We utilized the same interview guide with minor edits for it to make sense for both cohorts.

After the interview, participants completed a brief survey to assess demographic and professional (for clinicians and informatics experts) characteristics. Participants received $20 gift cards as remuneration for completing the interview and survey. Once the interviews were complete, the recordings were transcribed using a HIPAA-compliant transcription service and de-identified.

### Coding/analysis

Results are reported in accordance with the sociotechnical framework. Transcripts were coded using QSR NVivo 12 using a codebook developed by research team members (C.P., K.C.). The two team members (C.P. and K.C.), were supervised by a principal investigator and senior team member (M.P. and R.F.). They double-coded two transcripts and checked for inter-rater reliability to ensure a kappa > 0.8 and percent agreement > 95%. They discussed discrepancies, revised the codebook as needed, and double-coded seven more transcripts. Once inter-rater reliability was obtained a second time, the remaining 13 transcripts were coded independently. Demographic and professional characteristics of participants were summarized using means and standard deviations for continuous variables and counts and percentages for categorical variables.

## Results

Twenty-eight patients were approached, and ten (36%) were enrolled as we reached saturation. Seventeen clinicians were approached and twelve (71%) were enrolled. Table 1 displays the participant characteristics. Most patients (90%) and clinicians (67%) were white. Most patient participants had Stage I breast cancer (70%), and all patients had breast reconstruction after mastectomy. Six (50%) of the clinician participants were male, eight (67%) were physicians, and 54% had spent less than 10 years in practice.

**Table 1:** Participant Characteristics (N = 22)

Characteristics	Number (%) unless indicated
**Patients**	**N = 10 (100)**
**Age, years**	
Mean (SD, Range)	45.5 (7, 35–59)
**Gender**	
Female	10 (100)
**Race**	
White	9 (90)
Black/African-American	0 (0)
Native American/Alaskan Native	1 (10)
**Household income, US dollars**	
Less than 30,000	0 (0)
30, 000–60, 000	0 (0)
More than 60, 000	8 (80)
Prefer not to answer	2 (20)
**Breast cancer stage**	
Stage I	7 (70)
Stage II	1 (10)
Stage III	2 (20)
**Type of reconstructive surgery**	
Implant	4 (40)
Flap or tissue-based	6 (60)
**Timing of reconstructive surgery**	
Immediate	8 (80)
Delayed	2 (20)
**Clinicians**	**N = 12 (100)**
**Gender**	
Male	6 (50)
Female	6 (50)
**Race**	
White	10 (83.3)
Asian-American	1 (8.3)
Black/African-American	1 (8.3)
**Clinician Background**	
MD	8 (66.7)
PA (Physician Assistant)	4 (33.3)
**Years in practice (range)**	
Less than 10	7 (58.3)
10–20	1 (8.3)
21 or more	0 (0)
Missing response	4 (33.3)
**Geographic area of practice**	
Urban	4 (33.3)
Suburban	3 (25)
Missing response	5 (41.7)

Example quotes according to each sociotechnical dimension can be found in Table 2. Patient and clinician participants expressed that the existing tool could enhance communication during the encounter by providing background information on breast reconstruction choices and individualized risk. Patients and clinicians thought that having the patient use the tool at home or in the waiting room would prepare them for the breast reconstruction conversation during their surgical consultation. They felt that this timing would improve communication or clinical workflow. Some expressed concern that the time spent in the waiting room using the tool may not be sufficient for the patient to feel completely prepared for the conversation during the consultation.

Clinicians stated that they typically encounter CDS through a notification system embedded in the EHR. They felt that this strategy is not effective at engaging clinicians with patient-related information. Instead, clinicians would prefer the information to be included in the patient’s chart or the clinician’s EHR inbox prior to the consultation with the patient. Clinicians also expressed that it would be helpful to provide a tablet computer to patients when they are in the waiting room prior to their appointment so that they can complete the risk assessment and view the tool.

In terms of innovation content and human-computer interaction, patients and clinicians agreed that the tool contains helpful information, does not require much time for the patient to use, and fills gaps in knowledge in a methodical way. Tool navigation was acceptable to patients, although some expressed initial challenges in understanding how to progress through the tool or why the tool didn’t automatically update or repopulate when options were toggled on the risk assessment page. Patients also found the risk profile page helpful, yet a bit dense with information – both text and visuals – so that it took longer to read and digest.

**Table 2:** Example quotations according to sociotechnical dimensions, theme, and participant type

Sociotechnical Dimensions and Theme	Example Quotation: Patients	Example Quotation: Clinicians
**People**		
Patient Clinician Communication · Help shared decision making between patients and providers. (10/22 participants) · Prepare better for their first appointment (8/10 participants)	“If you have any questions, you’d be more prepared for the doctor visit or at least have an idea of what you want so when you’re in there with the doctor, the patient can be more confident and say what they want or what they think they want and then start the conversation there with the doctor.” – Patient 28, implant reconstruction	“I think this [BREASTChoice Tool] is very helpful. These are a lot of the questions that people ask me, and it might bring up a little more of an informed, a guided discussion with some of this stuff.” – Clinician 40
Preference for Timing of BREASTChoice Summary • Receive the patient’s BREASTChoice summary right before the patient is seen (6/12 participants) · Print or email the summary. (6/12 participants)	“The way I generally approach my clinic is one patient at a time kind of thing. If it’s [BREASTChoice summary] an inbox thing that I need to read before I go see the patient, as long as it’s there and it’s easily accessible and relatively condensed information, I think that that’s okay. I don’t think that’ll disrupt much of anything. It may help because now I know what the patient’s thinking a little bit before you even get in there.” – Clinician 41	N/A
	“Honestly, [receiving the summary] probably right before I see the patient. Not days in advance and not afterwards, but within that clinic day or immediately prior to walking in to have the conversation with the patient.” – Clinician 39	
**Workflow and Communication**		
Location to View Tool • Patients prefer to use the tool at home (7/10 participants) · Clinicians indicated that patients could also benefit from using the tool in the waiting room prior to their appointment. (5/12 participants)	“I think I would probably prefer at home because I feel like I could really take the time to go through it, and then if I had questions from, I would just write any of that down and then take that into my doctor.” – Patient 20, flap reconstruction	“There’s a lot of waiting when they come to our clinics, so I think it makes sense to give them some—these sorts of tools are perfect because it triggers their line of questioning when they’re finally seen, and also it minimizes their annoyance that we’re behind.” -Clinician 45
Timeline to Use Tool • Beneficial for patients to use the tool prior to their plastic surgery appointment (16/22 participants)	“I wish I would have seen this before my first appointment with the surgeon. I felt like my first plastic surgery appointment, I thought it was just to do what we’re doing right now, to go over my options, to learn about what I can do, but in fact, it was the opposite.” – Patient 6, flap reconstruction	N/A
**Organizational Policies and Culture**		
EHR Integration • Clinicians were unsure that EHR alerts were the best way to get the patient summary (3/12 participants)	N/A	“Someone’s got to get it [summary] to print it off and put it in my face or shoot me an email just before I see the patient. I’m going to be very honest when it comes to BPAs. I think we all just click dismiss. They’re nice, but I don’t think they’re the most effective. If there would be another way—will it be in the patient’s chart?” – Clinician 50
**Hardware and Technical Infrastructure**		
Tablet Computers • Helpful to provide patients with tablet computers to view the tool in the waiting room (5/12 participants)	N/A	“If they [patients] can get iPads or something like that or while in a waiting area for plastics, that’s something that they can fill out because I think it would be really helpful for the patients.” – Clinician 45
**Innovation Content**		
Design · Most liked the design and thought the color scheme was appropriate and the tool was user friendly (19/22 participants) · Some found the tool to be a little “text heavy” and preferred content be easier to read and digest (14/22 participants)	“I think it’s [BREASTChoice tool] very clear. It’s not too wordy. It’s got just the right amount of information. No. I think definitely it’s not too busy. Sometimes there’s so much on a screen that you don’t even know where to go.” – Patient 16, implant reconstruction	“I think it’s simple, straightforward. I think it’s easy to navigate. I think my overall impression is that I’ve read a lot of words. I think if you could have some sort of way to relieve some of that, just the wordiness of it. This is all very, very important information, but I’m just not sure how many people are going to actually pay attention to all of these details, whereas if you have someone lay it for you or speak you through it or talk you through it, I think a lot of the comprehension may be a little bit better.” – Clinician 40
Diagram · Gave patients a better understanding of the reconstruction procedures (17/22 participants) · Diagrams could have been more beneficial to clinicians if they were less cartoon-like and more realistic (8/12 participants)	“I think this picture [of implants] is really good…for understanding really how it’s put in place…how the implant works.” – Patient 24, implant reconstruction “Just showing where they’re taking the tissue from, the skin and stuff that’s helpful because if you just said latissimus dorsi flap, nobody would know what you were talking about.” – Patient 16, implant reconstruction	“So I don’t think it’s—it’s not the best picture I’ve seen. And it’s very cartoonish. I guess I don’t love the drawings.” – Clinician 202
Layout of Content · Information presented in a way patients would be able to understand (17/22 participants)	N/A	“I think that the format it’s laid out in [BREASTChoice Tool] is actually really nice, just kind of how from the beginning to now with listing the delayed, immediate, and now you’re getting into the actual reconstruction itself with the implants. Again, I’m a big fan of this. It gives the pros and cons just bulleted and just more to the point.” – Clinician 48
Length of Tool · Length was manageable (20/22 participants)	N/A	“This is a topic that I think the women will be very engaged in. It’s [length] not overwhelming at all, and it’s presented in nice, short packets that I think are very manageable.” – Clinician 37
**Human Computer Interaction**		
Tool Navigation · Easy to navigate (20/22 participants) · Appreciated the ability to travel back and forth between pages (13/22 participants) Risk Profile Navigation · Navigating the risk profile tool can be confusing due to the amount and layout of information on the page (10/22 participants) · Risk profile assessment illustrates the risks associated with different forms of reconstruction (6/10 participants)	“I like that, after each page, there’s an arrow to direct you to the next. I like that they’re not long pages either, which I think will be good for a lot of patients if they’re navigating this as an older group, just to keep it short and sweet because, sometimes, you just get so overwhelmed with information that you’re not actually absorbing anything because of all the emotions too.” – Patient 14, flap reconstruction “On this page, when you first start reading it, you have to scroll down a little bit to be able to see the other parts. When I first see it, I think it distracts me from the reading, and I look over to see what this is about or what this is for. What are all these people over here for? I think it’s a good visual. It’s just what shows up on your screen and having to scroll through. They may skip some of the reading or be distracted by it at first.” – Patient 23, flap reconstruction “I had no clue what my chances were of having any kind of infection or anything or tissue damage or anything like that, so I think that that it’s nice to know for people looking into it what their chances are of having it.” – Patient 7, implant reconstruction “Just reading top to bottom, it’s like, you have a risk level of X. Where’s my risk level coming from? It’s (risk profile page) a little hard to figure it out at first. If you don’t read everything there, then you might be confused on how it’s working.” – Patient 24, implant reconstruction	N/A

## Discussion

We conducted qualitative interviews with patients and clinicians guided by a sociotechnical framework to evaluate BREASTChoice according to people, workflow and communication, organizational policies and culture, hardware and technical infrastructure, innovation content, and human-computer interaction. Consistent with the literature, which typically uses a minimum of 6 to 8 participants per cohort, we used a similar focused approach for our usability testing [1, 4].

Patients and clinicians thought that viewing the tool prior to the appointment would help the patient be better prepared for the breast reconstruction conversation during their surgical consultation. However, there may not be enough time to comprehensively review the tool in the waiting room, especially the risk profile page.

Additional modifiable factors to the workflow and content that would improve the tool’s utility and sustainability may include delivering the information via the EHR inbox to the clinician prior to the consultation with the patient, providing a tablet computer in the waiting room for patients to use to view the tool, making some small changes to the user interface for page navigation, and providing a clearer explanation or layout of the risk profile section of BREASTChoice.

Strengths of this study included its multicenter design to evaluate implementation and sustainability potential across sites. While the two sites were located in the Midwest, it is a strength of the study that usability was assessed and deemed acceptable among patients and clinicians in distinct practice settings with different workflows and patient characteristics. We additionally used a sociotechnical framework to guide the usability evaluation of BREASTChoice according to end-users (both patients and clinicians), human-computer interaction, and contextual factors. We also included MDs and physician assistants to represent the full scope of end-user clinicians. We evaluated risk communication, and improvements that could be made in terms of communicating risk, which remains one of the most challenging – and impactful – aspects of delivering appropriate decision aids via CDS.

Limitations included the relatively young group of clinicians and patients with limited racial or ethnic diversity. As a result, we acknowledge the potential for bias in the thematic results based on a lack of diversity of the participant cohorts. We could have had a more robust recruitment plan to reach more patients. In addition, we uncovered a lack of experience with usability testing among clinicians and patients, some difficulty among participants in interpreting open-ended interview questions, and clinician saturation with CDS (often referred to as “alert fatigue”). In addition, we did not evaluate the tool according to system monitoring and measurement, a component of the sociotechnical framework, since the tool had not yet been implemented and we were not seeking to evaluate how it impacted the technical ecosystem. However, this will be an important aspect of our ongoing evaluation once the tool goes live across sites.

## Conclusions

This work demonstrated a high level of usability and potential for sustainability of BREASTChoice use among patients and clinicians. Our next step is to implement the tool across these two sites after integrating the suggested changes to workflow and content that we uncovered with this analysis. It is critical to conduct usability assessments such as these prior to CDS implementation to ensure success of the tool at the point-of-care.

## Appendix

### Summary table

*What was already known on the topic*.

- Clinical decision support (CDS) tools can facilitate point-of-care decision-making.
- CDS tools have been successfully tested to improve a broad range of health outcomes.
- Our previous work has demonstrated the efficacy of a CDS tool to support women’s decisions about post-mastectomy breast reconstruction (BREASTChoice).
- BREASTChoice has the potential to facilitate shared decision-making, improve workflow, and enhance the efficiency of a breast reconstruction consultation.

*What this study adds to our knowledge*.

- We demonstrate a high level of usability and potential for sustainability of BREASTChoice use among patients and clinicians.
- Our next step is to implement the tool across these two sites after integrating the suggested changes to workflow and content that we uncovered with this analysis.
- Usability assessments such as these are critical to conduct prior to CDS implementation to ensure success of the tool at the point-of-care.

## Electronic supplementary material

Below is the link to the electronic supplementary material.

Supplementary Material 1

## Data Availability

De-identified study data may be shared upon request by contacting the corresponding author via email: randi.foraker@wustl.edu.

## Abbreviations

CDS: Clinical decision support
EHR: Electronic health record
DCIS: ductal carcinoma in situ

## References

[1] Chrimes D, Kitos NR, Kushniruk A, Mann DM (2014). Usability testing of avoiding diabetes thru Action Plan Targeting (ADAPT) decision support for integrating care-based counseling of pre-diabetes in an electronic health record. Int J Med Informatics.

[2] Mann DM, Palmisano J, Lin JJ (2016). A pilot randomized trial of technology-assisted goal setting to improve physical activity among primary care patients with prediabetes. Prev Med Rep Dec.

[3] Foraker RE, Shoben AB, Kelley MM (2016). Electronic health record-based assessment of cardiovascular health: the stroke prevention in healthcare delivery environments (SPHERE) study. Prev Med Rep.

[4] Boateng J, Lee CN, Foraker RE (2021). Implementing an electronic clinical decision support Tool Into Routine Care: a qualitative study of Stakeholders’ perceptions of a Post-Mastectomy breast Reconstruction Tool. MDM Policy Pract Jul-Dec.

[5] Politi MC, Lee CN, Philpott-Streiff SE (2020). A Randomized Controlled Trial evaluating the BREASTChoice Tool for personalized decision support about breast Reconstruction after Mastectomy. Ann Surg Feb.

[6] Greenberg JK, Otun A, Nasraddin A (2021). Electronic clinical decision support for children with minor head trauma and intracranial injuries: a sociotechnical analysis. BMC Med Inform Decis Mak May.

