# Supplementary material for: Using the sociotechnical model to conduct a focused usability assessment of a breast reconstruction decision tool
Source: BMC Med Inform Decis Mak. 2023 Jul 28;23:140. doi: 10.1186/s12911-023-02236-x (PMC10375746; doi:10.1186/s12911-023-02236-x)
Supplement: Supplementary file 1 — Supplementary Material 1 [file 12911_2023_2236_MOESM1_ESM.docx]

**Appendix A.**

BREASTChoice Interview Guide

Thank you for filling out the questionnaire. Before you leave, I would love to talk briefly about your overall impression of BREASTChoice.

1. Right now how do you feel about BREASTChoice after the session today?
2. Were there problems that you encountered with the website?
3. Was there anything in BREASTChoice that you think worked well?
4. What do you think of the layout of the website?
5. What do you think of the design?
6. Was it easy or difficult to navigate through the website?
   1. Why did you find easy to navigate? Or
   2. What did you find difficult to navigate?
7. What did you think of the length of the tool?
   1. Were any sections too long or too short?
8. For patients: When do you think would be the best time for patients to receive this tool?
9. For clinicians: How best could this fit into your workflow?
10. What do you think of the risk predication model used in the tool?
11. Now that we are finished with that part [of the session], do you have additional general thoughts about BREASTChoice?
12. Do you have any other feedback, concerns, or questions?
